# Large uterine fibroid causing DVT and PE: Successful management with mechanical aspiration thrombectomy and hysterectomy: A case report and literature review

**DOI:** 10.1097/MD.0000000000040862

**Published:** 2024-12-06

**Authors:** Asfia Qammar, Sandesh Raja, Adarsh Raja, Aayush Chaulagain, Poupak Moshayedi, Cara East

**Affiliations:** a Baylor Scott & White Heart and Vascular Hospital, Dallas, TX; b Dow Medical College, Dow University of Health Sciences, Karachi, Pakistan; c Shaheed Mohtarma Benazir Bhutto Medical College Lyari, Karachi, Pakistan; d Patan Academy of Health Sciences, Lalitpur, Nepal.

**Keywords:** deep vein thrombosis, pelvic vein compression, pulmonary embolism, thromboembolic complications, uterine leiomyoma

## Abstract

**Rationale:**

Uterine leiomyomas, though commonly benign, can occasionally lead to serious complications, including deep vein thrombosis (DVT) and pulmonary embolism (PE). This study aims to highlight the uncommon yet serious association between large uterine leiomyomas and thromboembolism, which is often overlooked in patients without traditional risk factors. It emphasizes the need for awareness, early diagnosis, and timely intervention to prevent complications in patients presenting with unexplained symptoms and pelvic masses.

**Patient concern:**

A 38-year-old gravida 5, para 2 woman presented to the emergency room with left lower extremity swelling, pain, and discoloration, accompanied by dyspnea. She had no prior history of DVT or PE and did not have any known risk factors for venous thromboembolism.

**Diagnosis:**

The patient was diagnosed with DVT and PE, confirmed by venous duplex ultrasound and abdominal and pelvic computed tomography, which revealed thrombus extension to the left iliac vein. Chest computed tomography angiography confirmed a partially occlusive thrombus in the pulmonary arteries.

**Intervention:**

The patient underwent mechanical aspiration thrombectomy, followed by placement of a left iliac stent. Anticoagulation therapy with heparin was initiated post-thrombectomy. On the third day, a right supracervical hysterectomy was successfully performed. After surgery, anticoagulation was continued with heparin, and the patient was later discharged on apixaban for ongoing therapy.

**Outcomes:**

The patient made full recovery with no recurrence of thromboembolic events at 11 months posttreatment.

**Lessons:**

This case highlights the rare but serious complication of DVT and PE in patients with uterine leiomyomas. Timely intervention with thrombectomy, stent placement, and hysterectomy was effective in resolving the thromboembolic events.

## 1. Introduction

Uterine leiomyomas, the most common pelvic neoplasms in females, are found in approximately 50% to 60% of women, with prevalence increasing to 70% by the age of 50. Originating as monoclonal growths from the smooth muscle cells of the myometrium, these tumors can manifest as subserosal, submucosal, or intramural formations. Menorrhagia stands as the predominant symptom, while pelvic pain or pressure is less frequently reported. The proliferation of these tumors is regulated by estrogen, growth hormone, and progesterone.^[1,2]^

Although uterine leiomyomas are the most common neoplasms in women of reproductive age, they rarely cause acute complications. When such complications do occur, they can lead to significant morbidity and mortality, including thromboembolism, torsion of subserosal pedunculated leiomyomas, urinary retention, and vaginal or intraperitoneal hemorrhage. A rare but documented cause of thromboembolism is uterine leiomyoma, which, by exerting pressure on the pelvic veins, can precipitate deep vein thrombosis (DVT) and subsequent pulmonary embolism (PE) in certain patients. Only a limited number of reports elucidate this correlation in patients without additional venous thromboembolism risk factors.^[3–25]^

We detail the clinical, pathological, and radiological findings from a case involving a substantial uterine leiomyoma that induced pelvic venous stasis, culminating in DVT and PE, which was managed without complications. Additionally, we conducted a comprehensive literature review of cases documenting DVT and PE associated with uterine fibroids. Comprehensive verbal and written informed consent for the publication of this study was obtained from the patient.

## 2. Case presentation

A 38-year-old gravida 5, para 2, nonsmoking married woman presented to the emergency room with complaints of left lower extremity swelling, pain, and discoloration for the past 2 days, accompanied by dyspnea that began simultaneously with the leg symptoms. She reported no history of DVT or PE. She has not undergone surgery in the past 2 years, has not traveled by air in the past year, and uses an intrauterine device.

**Figure 1.**
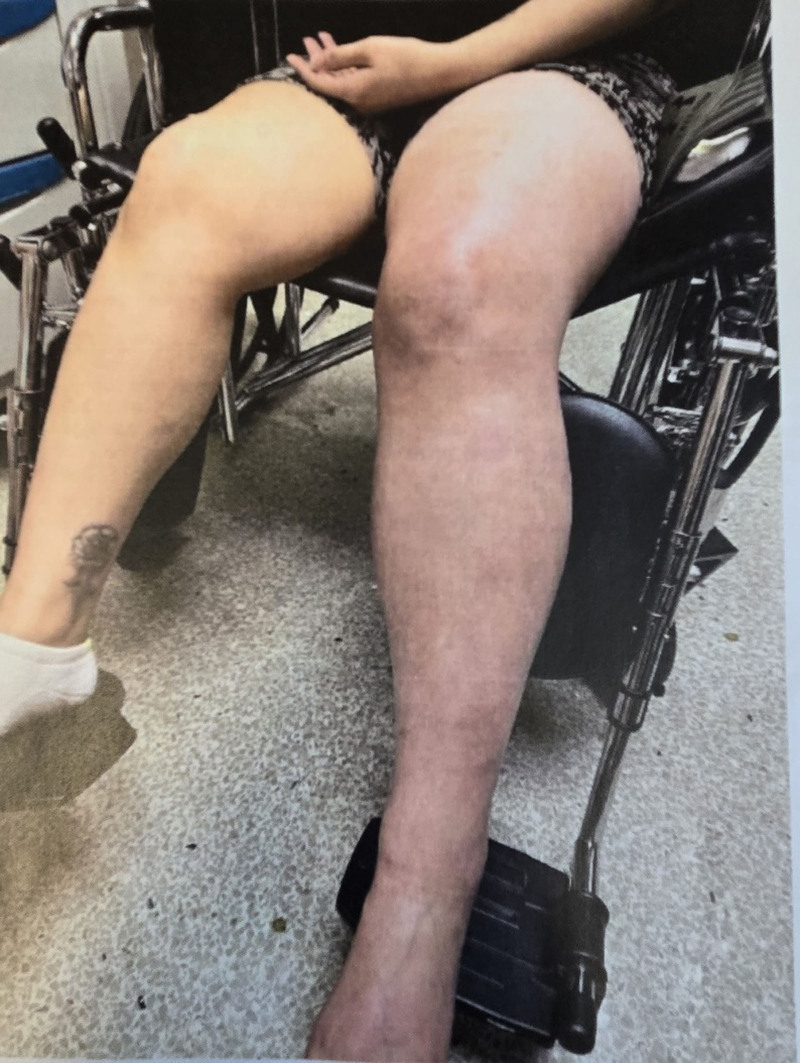
Inflamed and cyanosed left leg.

Physical examination revealed a well-nourished woman who was not acutely uncomfortable and appeared pale, with a body mass index of 32.7 kg/m². She reported sleeping for 7 hours. Examination findings included normal auditory and visual assessments, slightly warm skin to the touch, and slight pallor. Vital signs were: temperature 37.3 °C, blood pressure 119/76 mm Hg, pulse 113 bpm, respiratory rate 18 breaths per minute, and oxygen saturation normal at 100% on room air. There were signs of inflammation, edema, and cyanosis in the left lower limb, as shown in Figure 1. No significant findings were noted on the systemic examination, including the abdomen, which was neither tender nor distended.

**Figure 2.**
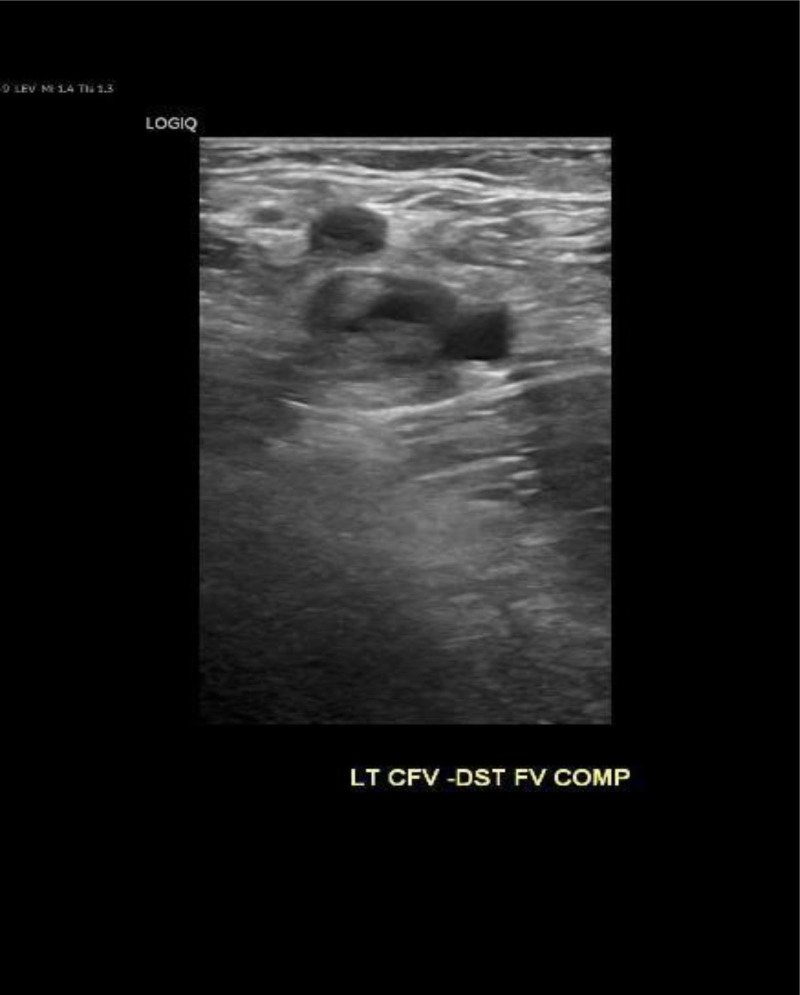
Duplex ultrasound showing DVT. DVT = deep vein thrombosis.

**Figure 3.**
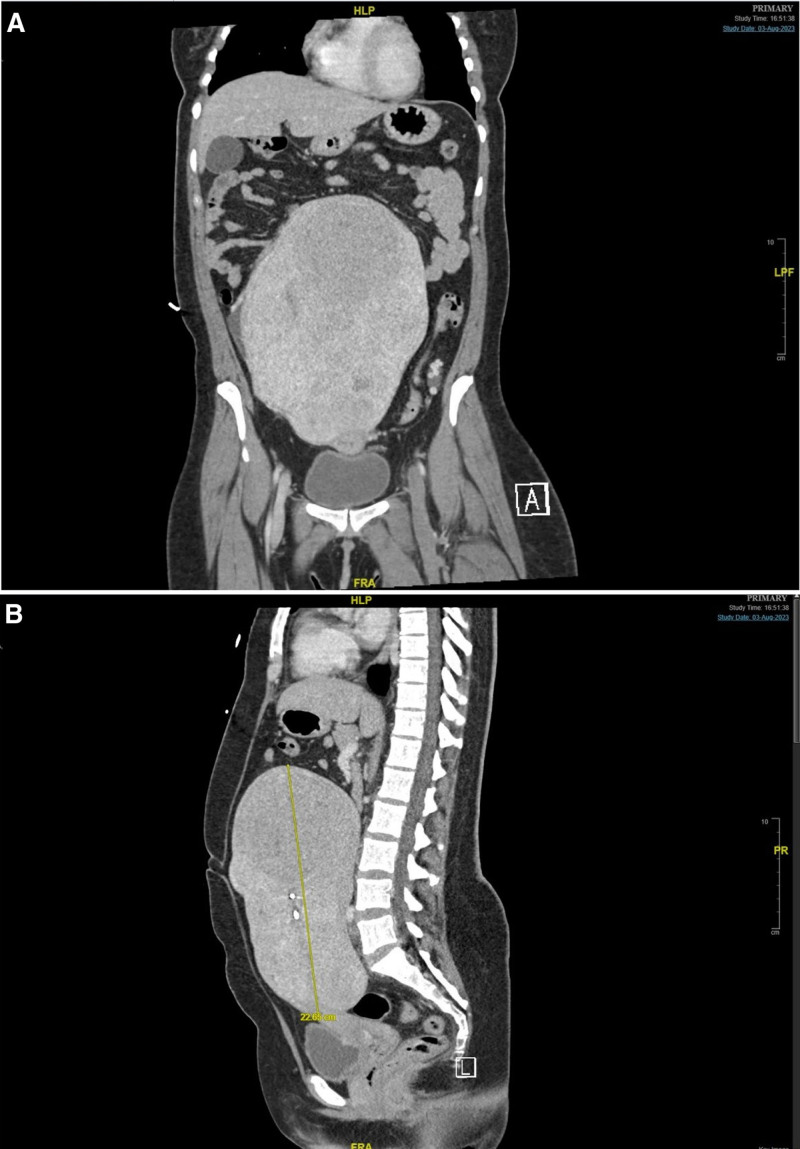
Abdominal and pelvic CT scans with contrast showing a large fibroid compressing the veins. (A) Coronal view; (B) sagittal view. CT = computed tomography.

**Figure 4.**
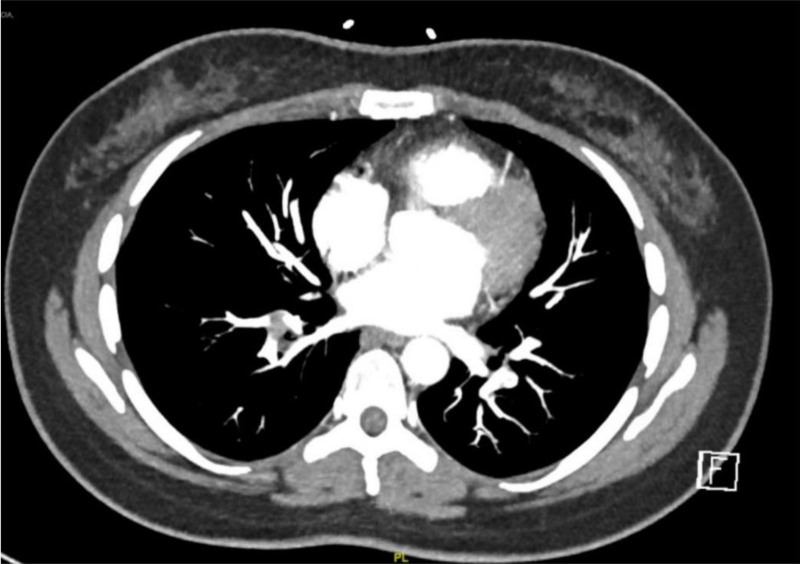
Chest CTA showing bilateral PE. CTA = computed tomography angiography, PE = pulmonary embolism.

Hematologic testing revealed anemia, while renal function tests showed slightly elevated urea and creatinine levels. Coagulation factor profiles and EKG results were unremarkable. A venous duplex ultrasound of the left lower extremity indicated complete occlusion of major veins, including the common femoral vein, greater saphenous vein, popliteal vein, and superficial femoral vein (Fig. 2). An abdominal and pelvic computed tomography scan with contrast showed deep vein thrombosis in the left lower extremity extending to the left iliac vein, where it passes adjacent to the region (Fig. 3). Subsequent chest computed tomography angiography (CTA) revealed a partially occlusive acute thrombus arising from the distal segments of the main pulmonary artery bilaterally (Fig. 4).

**Figure 5.**
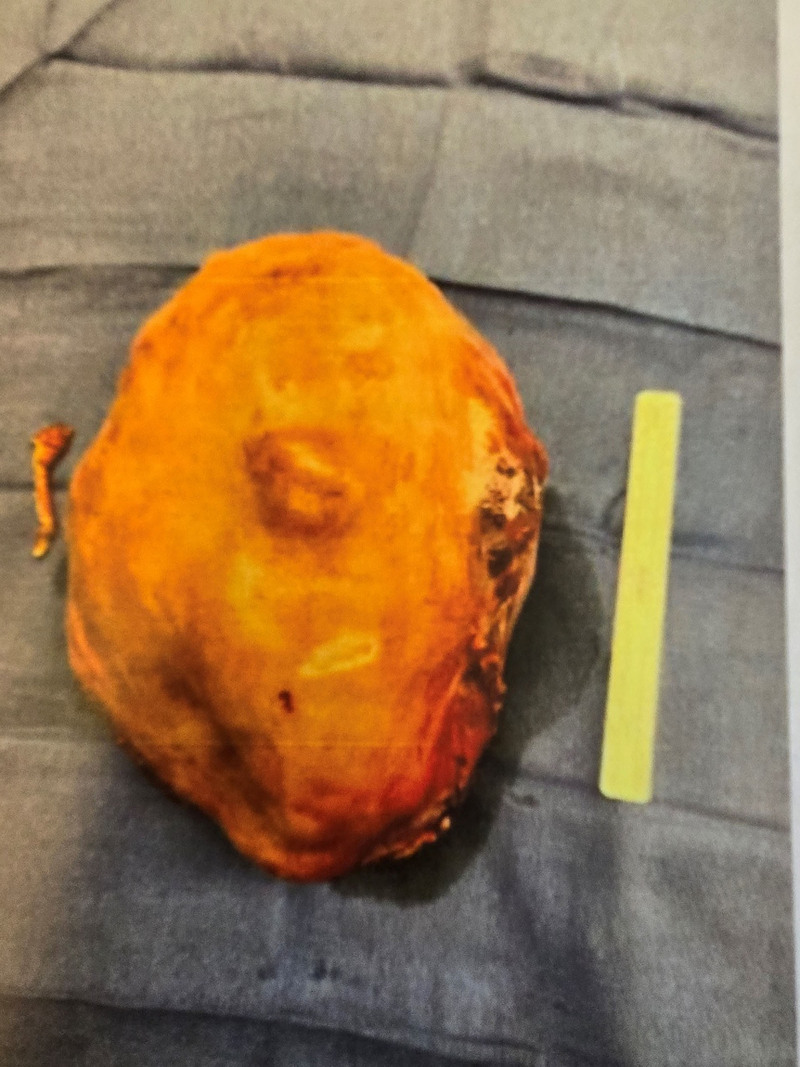
Gross examination of the leiomyoma.

As a result of the PE, the patient was administered 6.5 ml of heparin sodium porcine, containing 6500 IU. Perioperative care included mechanical aspiration thrombectomy followed by left iliac stent placement. After assessing her postoperative condition, heparin porcine 1000 units/mL was administered every 6 hours as needed, based on current Anti Factor X levels. The patient was started on 325 mg of ferrous sulfate tablets once daily for microcytic anemia. On the third day of admission, exploratory laparotomy was performed, followed by right supracervical hysterectomy and salpingectomy. The procedure was uneventful. Gross examination of the leiomyoma revealed a uterus measuring 21.0 × 17.0 × 11.5 cm, distorted with multiple tan-white, well-circumscribed nodules ranging from 0.4 to 11 cm (Fig. 5). The smallest nodule weighed 438 grams, and the largest weighed 2231 grams. Notably, the left fallopian tube was not visible, while the right fallopian tube contained numerous blood clots and a 0.4 cm cyst at the fimbriated end. Once an INR of 2.0 to 3.0 was achieved, the patient was discharged on apixaban 5 mg, 2 tablets daily.

This report highlights the successful management of DVT and PE using mechanical aspiration thrombectomy followed by stent placement. Anticoagulation therapy was initiated post-thrombectomy, and the patient’s recovery was closely monitored. After an uneventful supracervical hysterectomy, follow-up tests over 4 months showed no recurrence of thromboembolic events. At 11 months, the patient remained in good health, with no signs of thromboembolism, demonstrating the effectiveness of timely interventions and a favorable long-term outcome. However, it is important to recognize the limitations of this study. Aspiration mechanical thrombectomy for DVT requires specialized equipment and skilled operators, which may not be accessible in all settings. Although it has a lower bleeding risk than systemic thrombolysis, risks of venous injury and other complications persist. Additionally, its long-term benefits, especially in preventing post-thrombotic syndrome, require further study to confirm efficacy.

## 3. Discussion

Exceedingly common yet typically benign and asymptomatic, uterine fibroids can precipitate various gynecologic and obstetric complications, including the rarer but significant occurrences of DVT and PE. Upon diagnosis, patients with extensive thromboembolic disease secondary to uterine fibroid compression necessitate a multifaceted therapeutic approach. This approach includes both thrombus management and subsequent fibroid excision, the latter being critical to diminishing the likelihood of thrombus recurrence. While hysterectomy remains the gold standard treatment for thrombosis associated with uterine fibroids, myomectomy offers a conservative alternative for those wishing to preserve fertility, often complemented by inferior vena cava (IVC) filters and anticoagulant agents. Despite advancements in this field, the optimal therapeutic strategy, including thrombolysis and catheter-based interventions, remains indeterminate. It is crucial to consider patient preferences, age, quality of life, and comorbid conditions when determining the appropriate therapy.^[26–28]^

Virchow’s triad outlines the pathogenesis of thromboembolic disease, suggesting that venous thromboembolism (VTE) arises from 3 interrelated factors: hypercoagulability, whether systemic or localized; venous stasis; and endothelial injury to the vessel wall. The proposed mechanism linking large uterine leiomyomas to thromboembolic events involves extrinsic mechanical compression of adjacent anatomical structures, including the pelvic venous system, particularly in women without other known risk factors for thromboembolism. This compression induces venous stasis, which subsequently precipitates thrombosis.^[26]^

A comprehensive literature search was undertaken utilizing PubMed database, spanning from their inception through 2024. The search employed the following keywords: “thrombosis,” “thromboembolic disease,” “pulmonary embolism,” “uterine myoma,” “uterine fibroid,” and “leiomyoma.” Additionally, reference lists from selected articles were meticulously examined for further relevant studies. Our review identified 23 articles encompassing 29 cases of DVT and PE associated with uterine leiomyoma. The clinical presentations varied, featuring symptoms such as dyspnea, limb swelling, and diverse venous thrombosis patterns leading to PE. Predominantly, therapies included anticoagulant treatments and total abdominal hysterectomy, with some cases receiving adjunctive interventions such as IVC filters, thrombolysis, and embolectomy. In almost all cases, no other risk factors for VTE were present (Table 1).

**Table 1:** Literature review.

Study and year	No. of cases	Age (years)	Presentation	Diagnosis	DVT/PE	Uterine fibroid
Ogawa N et al (1992)^[24]^	1	49	Dyspnea	Left external iliac vein compression + PE	Embolectomy + thrombectomy	Hysterectomy
Tanaka H et al (2002)^[10]^	1	46	Dyspnea and dizziness on exertion	Thrombus in the right femoral vein + PE	Anticoagulant therapy	Hysterectomy
Boos CJ et al (2005)^[25]^	1	46	Acute shortness of breath and pre-syncope	Compressed IVC + PE	Anticoagulant therapy	Uterine artery embolization
Falcone M et al (2005)^[7]^	1	39	N/A	Lower limb DVT + PE	Thrombolysis + anticoagulant therapy	Total hysterectomy
Bonito M et al (2007)^[17]^	1	49	Left thigh pain and dyspnea	Compressed pelvic veins + bilateral PE	Anticoagulant therapy + IVC filter	Total abdominal hysterectomy
Nawaz et al (2008)^[11]^	1	41	Right lower limb swelling	Thrombosis of right proximal superficial femoral, right popliteal, right posterior and anterior tibial vein, and right lesser saphenous vein + right PE	Anticoagulant therapy	Total abdominal hysterectomy
Unosawa S et al (2009)^[6]^	1	53	Dyspnea	Compressing pelvic veins + bilateral PE	IVC filter + embolectomy	Hysterectomy
Huffman-Dracht HB et al (2010)^[18]^	1	37	Left lower extremity swelling and pain	Thrombosis of popliteal vein up to the mid-femoral vein + bilateral PE	Anticoagulant therapy + IVC filter	Hysterectomy
Kurakazu M et al (2012)^[15]^	1	40	Acute respiratory distress	Compressed left lateral iliac vein and bilateral common iliac veins + bilateral PE	Thrombolysis + anticoagulation therapy	Total abdominal hysterectomy
Lee D et al (2012)^[14]^	1	44	Right leg swelling	Thrombosis of the proximal popliteal vein up to the right common iliac vein + right PE	Percutaneous mechanical thromboembolectomy + thrombolysis + anticoagulant therapy	Total hysterectomy
Riat et al (2012)^[8]^	1	51	N/A	Left iliac vein thrombosis + PE	N/A	N/A
Rosenfeld H et al (2012)^[9]^	1	44	At autopsy (died due to massive PE)	Thrombosis of the left calf + bilateral PE	N/A	N/A
Srettabunjong S et al (2013)^[4]^	1	46	Dyspnea	Thrombosis of both lower limb veins + bilateral PE	N/A	N/A
Anuradha T et al (2014)^[21]^	1	33	Chest pain and shortness of breath	IVC thrombosis + PE	Anticoagulant therapy	Total abdominal hysterectomy
Fernandes FL et al (2014)^[20]^	1	29	Acute onset dyspnea, cough and pleuritic pain	Right iliac vein compression + bilateral PE	Anticoagulant therapy	Total hysterectomy
Khademvatani K et al (2014)^[16]^	1	42	Dyspnea and pleuritic chest pain	Thrombosis of popliteal and superficial femoral veins of left leg + left PE	Thrombolysis + anticoagulation therapy	Myomectomy
Podduturi V et al (2014)^[3]^	1	57	Progressive nonproductive cough and dyspnea	Thrombi in bilateral posterior tibial veins and deep gastrocnemius veins + bilateral PE	N/A	N/A
Brewer MB et al (2015)^[22]^	1	45	Left lower extremity edema	Left lower extremity venous thrombosis, right common iliac venous thrombosis + PE	IVC filter + left lower extremity venous thrombectomy	Total abdominal hysterectomy
Satti MA et al (2016)^[12]^	3	47	Left leg swelling and pain	Thrombosis of left common femoral vein, femoral, profunda femoral veins, and popliteal and tibial peroneal veins + left PE	Anticoagulant therapy + IVC filter	N/A
		60	Dyspnea and left lower back pain	Thrombosis of bilateral lower extremity veins + left PE	Anticoagulant therapy + IVC filter	Total abdominal hysterectomy
		41	Vaginal bleeding, dizziness, and shortness of breath	Died awaiting surgery due to massive PE	N/A	N/A
Nakamura S et al (2018)^[13]^	1	42	Dyspnea	Thrombus extending from her right ovarian vein into IVC + bilateral PE	Anticoagulant therapy	Transvaginal myomectomy
Lacharite-Roberge AS et al (2019)^[5]^	5	48	Exertional dyspnea, lower extremity swelling	Compression of IVC, both common iliac veins + PE	Pulmonary thromboendarterectomy	Supra-cervical abdominal hysterectomy
		53	Exertional dyspnea, lower extremity swelling	Compression of the right external iliac vein + PE	Pulmonary thromboendarterectomy	N/A
		35	Exertional dyspnea, lower extremity swelling	Compression of the right and left external iliac veins + PE	Pulmonary thromboendarterectomy	N/A
		53	Exertional dyspnea, lower extremity swelling	Compression of the IVC + PE	Pulmonary thromboendarterectomy	Total abdominal hysterectomy
		57	Exertional dyspnea, lower extremity swelling	Compressing the left common iliac vein + PE	Pulmonary thromboendarterectomy	N/A
Nartey Y et al (2020)^[23]^	1	41	Sudden onset chest pain	Thrombosis of the right femoral and popliteal veins + bilateral PE	Anticoagulant therapy	Total abdominal hysterectomy
Brown D et al (2024)^[19]^	1	33	Recurrent urinary retention	Bilateral external iliac veins compression + bilateral PE	Anticoagulant therapy	N/A

DVT = deep vein thrombosis, IVC = inferior vena cava, PE = pulmonary embolism.

No standardized protocols exist for managing uterine fibroids with DVT and PE. Brewer et al recommend a multifaceted approach, including immediate anticoagulation or thrombolysis/thrombectomy for extensive DVT, and emphasize fibroid excision to reduce DVT recurrence.^[22]^ Large uterine fibroids with extensive DVT may also be managed with immediate anticoagulation followed by uterine artery embolization to reduce uterine size, facilitating resolution of the DVT and subsequent total abdominal hysterectomy with a successful outcome.^[29]^ Conversely, Fletcher et al highlight the debate over anticoagulant use in menorrhagia due to bleeding risks and stress the need for anticoagulation reversal before surgery to prevent hemorrhage, while recommending mechanical compression stockings or low molecular weight heparin during surgery to avoid venous stasis complications.^[30]^ Secondary prevention of PE can involve retrievable IVC filters to prevent thrombus migration, though retrieval fails in up to 15% of cases.^[31]^

In the present case, thrombectomy was performed with mechanical aspiration using the Penumbra Bolt device, resolving thrombosis. Due to the patient’s parity and the large size of the fibroid, a hysterectomy was performed. CTA of the chest was utilized for diagnosing the suspected pulmonary embolism. It was concluded that the DVT and the subsequent PE developed due to the direct compression caused by the mass. Mechanical aspiration thrombectomy, along with anticoagulation therapy and supracervical hysterectomy, has proven to be a successful intervention with no complications in the past year. However, questions remain regarding whether mechanical thrombectomy is superior to thrombolysis and the optimal, safe treatment strategy to reduce leiomyoma size in such patients. Further research and experimental trials are necessary to clarify these issues.

## 4. Conclusion

DVT and subsequent PE due to pelvic vein compression are rare but significant complications of uterine leiomyoma, with few cases reported. For such cases, management can include mechanical aspiration thrombectomy, stent placement, and subsequent anticoagulation therapy and hysterectomy. CTA of the chest can be essential for diagnosing suspected high-risk PE. Given the rarity of this condition, early recognition depends on thorough clinical evaluation, especially in women presenting with an abdominal mass and pelvic pressure without a clear common cause for their symptoms. While universal screening for VTE in all patients with large uterine leiomyoma is not currently recommended, compression ultrasound may be considered for those with additional VTE risk factors. Prophylactic anticoagulation could be beneficial in reducing VTE risk in patients with large uterine leiomyoma prior to surgery, unless contraindicated by vaginal bleeding.

## Author contributions

**Conceptualization:** Asfia Qammar.

**Project administration:** Asfia Qammar.

**Supervision:** Asfia Qammar, Cara East.

**Writing – original draft:** Asfia Qammar, Sandesh Raja, Adarsh Raja, Aayush Chaulagain, Poupak Moshayedi, Cara East.

**Writing – review & editing:** Asfia Qammar, Sandesh Raja, Adarsh Raja, Aayush Chaulagain.
